# The Dual Role of PDCD10 in Cancers: A Promising Therapeutic Target

**DOI:** 10.3390/cancers14235986

**Published:** 2022-12-03

**Authors:** Jingdian Liu, Kai Zhao, Sisi Wu, Chaoxi Li, Chao You, Junwen Wang, Kai Shu, Ting Lei

**Affiliations:** Department of Neurosurgery, Tongji Hospital Affiliated to Tongji Medical College, Huazhong University of Science and Technology, Wuhan 430030, China

**Keywords:** PDCD10, cancers, apoptosis, metastasis, tumor microenvironment

## Abstract

**Simple Summary:**

As an adaptor protein, PDCD10 is involved in regulation of diverse biological processes by interacting with multiple molecules. Recently, growing amounts of studies have focused on function of PDCD10 in cancers. However, PDCD10 seems to have a dual role (either pro- or anti-tumor effects) in various cancer types, which may depend on cell/tissue specificity with different cellular interactors. In this review, we provided an overview of the structure and molecular functions of PDCD10, and summarized the knowledge of the dual role of PDCD10 in cancers, with a view to future development and application of PDCD10 as a clinical therapeutic target in cancers.

**Abstract:**

Programmed cell death 10 (PDCD10) was initially considered as a protein associated with apoptosis. However, recent studies showed that PDCD10 is actually an adaptor protein. By interacting with multiple molecules, PDCD10 participates in various physiological processes, such as cell survival, migration, cell differentiation, vesicle trafficking, cellular senescence, neurovascular development, and gonadogenesis. Moreover, over the past few decades, accumulating evidence has demonstrated that the aberrant expression or mutation of PDCD10 is extremely common in various pathological processes, especially in cancers. The dysfunction of PDCD10 has been strongly implicated in oncogenesis and tumor progression. However, the updated data seem to indicate that PDCD10 has a dual role (either pro- or anti-tumor effects) in various cancer types, depending on cell/tissue specificity with different cellular interactors. In this review, we aimed to summarize the knowledge of the dual role of PDCD10 in cancers with a special focus on its cellular function and potential molecular mechanism. With these efforts, we hoped to provide new insight into the future development and application of PDCD10 as a clinical therapeutic target in cancers.

## 1. Introduction

In 1999, a novel gene was identified in TF-1 cells, a human premyeloid cell line, by scientists at the Laboratory of Medical Immunology of Beijing Medical University [1]. They found that the expression of the novel gene was markedly increased after the deprivation of the granulocyte macrophage-colony stimulating factor in TF-1 cells by means of the cDNA-RDA method, and the corresponding recombinant protein could inhibit the natural cell death of human embryonic kidney 293T cells, which suggested its role as an apoptotic inhibitory factor. Thus, they termed the gene TF-1 cell apoptosis-related gene-15 (TFAR-15), which subsequently became known as programmed cell death 10 (PDCD10).

The PDCD gene family contains at least 12 members, which were all initially considered to be associated with apoptosis [2]. Later, numerous researchers reported that these genes also have multifaceted roles in a diverse array of cellular processes in addition to apoptosis. For example, the PDCD1 gene encodes PD-1 which regulates the activation of T-lymphocytes and immune responses and has become a validated immunotherapeutic cancer target in the clinic [3,4]. Although PDCD10 was initially considered an apoptosis-related protein, recent evidence suggested that it is actually an adaptor protein. Through its dimerization domain and focal adhesion targeting (FAT) homology domain, PDCD10 can directly interact with multiple molecules and thus regulate their functions. PDCD10 has been shown to be involved in the regulation of cell junction, cell cycle, cell motility, stem cell differentiation, vesicle trafficking, cellular senescence, and gonadogenesis [5,6,7,8,9,10].

Cerebral cavernous malformation 3 (CCM3) is another well-known name for PDCD10. The mutation of CCM3 has been identified to be associated with familial cavernous malformations (CCM). Although Craig et al. first demonstrated that the mutation of CCM3 was responsible for CCM in 1998 [11], PDCD10 was not verified as the CCM3 gene until 2005 [12]. In CCM-based studies, PDCD10 was found to interact with CCM1 and CCM2 to regulate endothelial homeostasis by forming the CCM signaling complex (CSC) [13].

In addition, recent reports showed that PDCD10 was also involved in many other diseases including myocardial ischemia-reperfusion injury, Alzheimer’s disease, and ankylosing spondylitis [14,15,16]. More importantly, over the past few decades, growing evidence has suggested that aberrant expressions or mutations of PDCD10 are extremely common in various solid cancers and hematological malignancies. The dysfunction of PDCD10 has been strongly implicated in oncogenesis and tumor progression. However, the updated data tend to indicate that PDCD10 plays a dual role (either pro- or anti-tumor effects) in various cancers, depending on cell/tissue specificity with different cellular interactors. In this review, we aimed to summarize the knowledge of the dual role of PDCD10 in cancers with a special focus on its cellular function and potential molecular mechanism. With these efforts, we hoped to provide new insight into the future development and application of PDCD10 as a clinical therapeutic target in cancers.

## 2. Structure, Interactors, and Regulators of PDCD10

### 2.1. The Structure of PDCD10

The human PDCD10 gene is mapped to chromosome 3 (3q26.1), contains 7 coding and 3 noncoding exons, and encodes 25 kDa proteins. As one of the PDCD gene family members, PDCD10 is highly evolutionarily conserved and is ubiquitously expressed in various tissues and cell types [2]. However, bioinformatics analysis revealed no overt sequence or structure similarity among PDCD gene family members, suggesting that each individual has unique functional properties [2,17].

**Figure 1:**
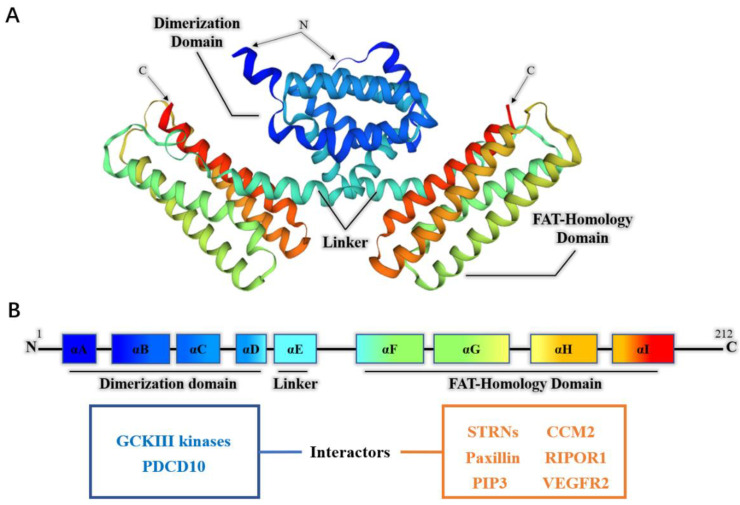
The structure and interactors of PDCD10: (**A**) the crystal structure of homodimerized PDCD10 and (**B**) the model of amino acid sequence, domains, and interactors of PDCD10.

As a protein of 212 amino acids, human PDCD10 is composed of 2 main domains folded from 9 alpha helices (αA-αI) (Figure 1). The dimerization domain of PDCD10 contains four alpha helices (αA-αD) at the N-terminal region, while the helices αF-αI make up a four-helical bundle which forms the FAT-homology domain at the C-terminal region [18]. Between the αD and αE, there is a hinge region around the lys-69 and lys-70 residues to allow the orientation of the two domains to vary by about 25° [18]. Within these domains, PDCD10 can bind to various interactors. For example, the dimerization domain, as the name suggests, is responsible for the homodimerization of PDCD10 [18]. This domain can also interact with germinal center kinase III (namely, GCKIII kinases, including STK24, STK25, and STK26) to form heterodimers [19,20,21,22,23]. The FAT-Homology domain was reported to be involved in the interactions with CCM2, striatins (STRNs), paxillin, phosphatidylinositol-3,4,5-trisphosphate (PIP3), RIPOR1, and VEGFR2 [18,21,24,25,26,27]. PDCD10 can also interact with KPNA2 and TRIM59 [28,29]. The structural features of PDCD10 suggest that it exerts functions as an adaptor protein. As discussed below, PDCD10 was reported to be involved in various physiological and pathological processes, and its interactors were thought to be responsible for the multifaceted nature of PDCD10.

### 2.2. The Interactors of PDCD10

#### 2.2.1. The Interactors of PDCD10 in Physiological Processes

As one of the core components of the striatin-interacting phosphatase and kinase (STRIPAK) complex, one of the most critical functions of PDCD10 is tethering GCKIII kinases to the protein backbone of the complex, STRNs [25,30,31,32]. In fact, STRNs are also known as B‴ regulatory subunits of protein phosphatase 2A (PP2A) [33]. In the STRIPAK complex, GCKIII kinases are recruited to STRNs with the help of PDCD10 and then are dephosphorylated and inactivated by PP2A [34], which indicates that PDCD10 can negatively regulate the activity of GCKIII kinases. For further details on the structure and function of the STRIPAK complex, the reader is referred to several excellent reviews [35,36,37]. Interestingly, PDCD10 and its homologs were detected only in animal systems [37], and pull-down experiments confirmed a direct interaction between STRNs and GCKIII kinases in fungi [38]. This implies that in mammalian systems, in addition to serving as a bridge in the STRIPAK complex, PDCD10 may have other effects on the regulation of GCKIII kinases. Exactly, PDCD10 was also reported to stabilize GCKIII kinases separately from the STRIPAK complex and regulate their localization [19,21,22,25,39], which seems to represent the positive regulation of GCKIII kinases mediated by PDCD10. For example, the knockdown of PDCD10 reduced the protein levels of all three GCKIII kinases by enhancing ubiquitylation and proteasome-dependent degradation [19]. PDCD10 also promoted the relocation of GCKIII kinases from the Golgi apparatus by binding to the C-terminal ARM domain of RIPOR1 [21].

Although PDCD10 was initially thought to be an anti-apoptotic protein, recent evidence suggested that PDCD10 could regulate cell survival and migration both positively and negatively [2,19,20,40,41,42,43,44,45,46,47]. Previous research has also shown that GCKIII kinases are involved in the regulation of the cell cycle, apoptosis, and migration [48]. Thus, the dual role of PDCD10 in cell survival and migration may be associated with its dual regulation of GCKIII kinases. The dual effect is also likely to depend in part on the subtype of GCKIII kinases. For example, by interacting with STK26, PDCD10 promotes cell survival via the ERK and ezrin/radixin/moesin (ERM) pathways [20,45]. In contrast, another study reported that PDCD10 could accelerate H_2_O_2_-induced cell apoptosis by stabilizing STK25 [46]. On the other hand, this interaction between PDCD10 and GCKIII kinases has also been demonstrated to be associated with the endothelial exocytosis of ANG-2, neutrophil degranulation, and AQP2 membrane targeting via regulating UNC13 family and ERM family proteins, suggesting that PDCD10 plays a role in vesicular trafficking [10,49,50].

In addition to GCKIII kinases, PDCD10 is also involved in the regulation of other molecules including PIP3, KPNA2, and so on. Evidence from computations and experiments confirmed that there is a direct interaction between PDCD10 and PIP3 [26]. As the phosphorylated product generated by PI3K, PIP3 can induce the activation of Akt. However, whether PDCD10 can positively or negatively regulate the PI3K-Akt pathway remains ambiguous. As a member of the nuclear transporter family, KPNA2 acts as a mediator during nuclear translocation [51]. A recent study revealed that PDCD10 can bind to KPNA2 and thus limits NF-κB nuclear translocation [28]. However, the domain of PDCD10 involved in this interaction is undefined.

#### 2.2.2. The Interactors of PDCD10 in Pathological Processes

CCM is a rare cerebrovascular disease, occurring predominantly in the central nervous system, which is characterized by irregular capillary-venous malformations and endothelial dysfunction [13]. CCM has been shown to be an autosomal dominant disease with incomplete penetrance, predominantly associated with mutations in 3 CCM genes (CCM1, CCM2, and CCM3/PDCD10) [11,52,53,54]. CCM1, CCM2, and PDCD10/CCM3 proteins do not share structure and sequence homology, but together they form CSC which acts as a protein complex that initiates multiple signaling cascades [13]. Several studies have reported that CSC is the key to inhibiting the activation of RhoA/ROCK and MEKK3, thus maintaining the endothelial barrier function to prevent CCM disease onset [55,56,57,58,59,60]. However, in CCM patients, mutations of CCM genes impair the function of CSC, which ultimately contributes to various CCM features including increased proliferation, aberrant migration, and endothelial-to-mesenchymal transition [13,61,62]. In addition, other researchers have reported that Notch signaling, intracellular reactive oxygen species levels, and autophagy are also regulated by CSC and participate in CCM pathogenesis [19,63,64,65,66].

In fact, compared to the CCM1 or CCM2 mutations, CCM patients with the PDCD10 mutation usually have these features including more severe symptoms, earlier onset, and more rapid progression [67,68], which has also been observed in animal models with PDCD10 deletion [69]. These results suggested that PDCD10 could contribute to CCM disease via other interactors (not just CSC). For example, only PDCD10 (but not CCM1 and CCM2) silencing increased the secretion of ANG-2 by interacting with GCKIII kinases, resulting in the dysfunctional endothelial junction during CCM development [49]. PDCD10 can also directly bind to VEGFR2 [27], which is a component of the VEGF signaling and is involved in the regulation of vascular development [70]. Loss of PDCD10 reduced VEGFR2 signaling in endothelial cells and led to defects in embryonic angiogenesis [27]. Moreover, loss of PDCD10 was shown to enhance the stability of paxillin, a hub for the regulation of focal adhesion, contributing to the impaired migration of pericytes and disruption of pericyte-endothelial cell interactions in CCM [18,71,72,73]. Moreover, a recent study reported that the knockout of PDCD10 can enhance caveolin-1-mediated endocytosis, inducing caveolae-Tie2 signaling, pericyte-endothelial cell disassociation, and CCM lesion formation [74]. Given that STRNs have a caveolin-binding domain, we speculate that this interaction between PDCD10 and caveolin-1 may be mediated by STRNs in the STRIPAK complex. In addition to these direct interactions between PDCD10 and other molecules, some pathways such as DLL4-Notch signaling, EphB4 signaling, and even the gut-brain axis were also reported to be regulated by PDCD10 in CCM [66,75,76], though how PDCD10 regulates these pathways needs further exploration. Altogether, the loss of PDCD10 in endothelial cells promoted proliferation, migration, and angiogenesis via regulating its interactors and related signaling, eventually leading to CCM.

In cancers, interactions between PDCD10 and its interactors appear more complex. In cancer-associated fibroblasts (CAFs), loss of PDCD10 promoted the nuclear translocation of YAP via interacting with paxillin, which contributed to tumor metastasis [5]. In contrast, in hepatocellular carcinoma (HCC), PDCD10 enhances the catalytic activity of PP2A through a direct or indirect physical interaction, which in turn promotes the dephosphorylation and nuclear translocation of YAP, leading to downstream oncogene expression [77]. The contradictory effect of PDCD10 on YAP activation suggests that the function of PDCD10 depends on cell/tissue specificity with different cellular interactors in cancers. On the other hand, PDCD10 was also reported to stabilize GCKIII kinases and facilitate their translocation to sites of actomyosin, which promotes cell contraction and the metastasis of cancer cells [78]. Given the negative regulation of GCKIII kinases mediated by PP2A, it seems paradoxical that PDCD10 not only enhances the catalytic activity of PP2A but also stabilizes GCKIII kinases in tumor cells. This suggests that the inactivation of GCKIII kinases mediated by PP2A may be affected by other components of STRIPAK, such as STRIP1/2 [78]. Moreover, the low STRNs/PDCD10 ratio in extracranial tissues may be another reason for this phenomenon. Previous reports showed that STRNs were most abundant in the central nervous system [79,80]. Thus, in extracranial tumors, it is tempting to speculate that the binding of PDCD10 to STRNs is saturated, and that the upregulated PDCD10 is more inclined to stabilize GCKIII kinases but not recruit GCKIII kinases to STRNs. The hypothesis is supported by much of the published literature, which reported that the expression of PDCD10 and GCKIII kinases are positively correlated with tumor grade and aggressiveness in breast cancer and prostate cancer, while expression of STRNs is reduced in aggressive breast cancer subtypes [78,81]. Some empirical evidence also showed that the depletion of PDCD10 or STRNs affects the sub-cellular localization of GCKIII kinases in an opposite manner [19,25]. Further details on the roles of PDCD10 in cancers will be discussed in the next section.

### 2.3. The Regulators of PDCD10

Ubiquitination is a major pathway of protein degradation. The tripartite motif (TRIM) family proteins usually have the activity of ubiquitin ligase and can selectively target ubiquitin-modified proteins [82]. As a member of the TRIM family, TRIM59 was reported to stabilize PDCD10 by preventing it from undergoing ubiquitination mediated by RNFT1 and subsequent p62/SQSTM1-selective autophagic degradation, which is currently the only known regulatory mechanism underlying PDCD10 degradation [29,83].

As a class of small non-coding RNAs, microRNAs (miRNAs) can modulate gene expression at the post-transcriptional level by inducing the degradation of mRNAs or inhibiting the translation of mRNAs through base complementary pairing. As summarized in Table 1, PDCD10 has been reported to be the target of multiple miRNAs and to act as an effector to exert their functions.

**Figure 2:**
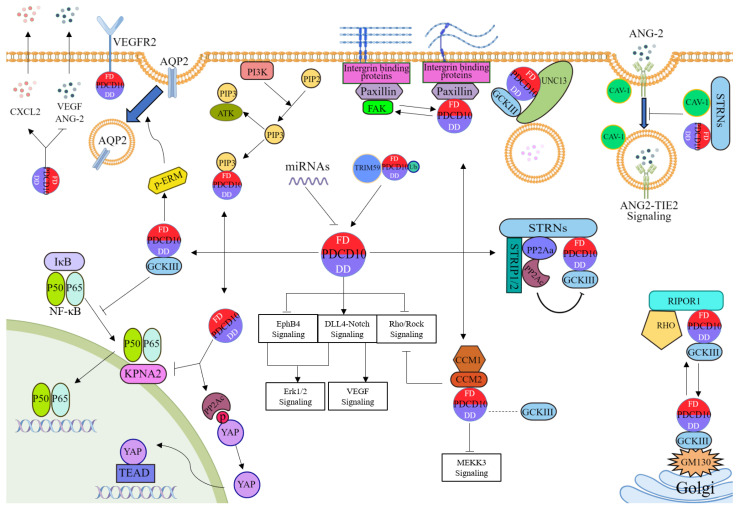
Interactors, regulators, and signaling of PDCD10. FD: FAT-Homology domain; DD: Dimerization domain. (By Figdraw. http://www.figdraw.com/ accessed on 26 November 2022).

Figure 2 summarizes the cellular interactors, regulators, and signaling of PDCD10.

## 3. The Dual Role of PDCD10 in Cancer

In the majority of cancer types, PDCD10 seems to promote oncogenesis. Upregulation of PDCD10 is observed in most human tumors according to the TCGA database. However, some studies discovered the anti-tumorigenic effects of PDCD10. Intriguingly, PDCD10 was even reported to have both tumor-suppressive and tumor-promoting effects in gliomas. The roles of PDCD10 in different cancer types were summarized in Table 2.

### 3.1. PDCD10 Exerts Pro-Tumorigenic Effects

#### 3.1.1. PDCD10 Promotes Survival and Self-Renewal of Tumor Cells

Lauenborg et al. reported that PDCD10 was constitutively expressed by malignant T-cell lines in 2010 [93]. Furthermore, PDCD10 silencing promoted the apoptosis and decreased proliferation of malignant T cells, which is the first to link PDCD10 to cancer [93]. PDCD10 was reported to be upregulated in HCC and cholangiocarcinoma, which was associated with aggressive clinicopathological features [77,104]. Furthermore, Sun et al. showed that the upregulation of PDCD10 promoted cell proliferation and tumor growth in HCC by increasing the PP2A activity and nuclear translocation of YAP [77]. Moreover, upregulation and the pro-survival/proliferative effects of PDCD10 were also demonstrated in non-small cell lung cancer (NSCLC), bladder cancer, ovarian cancer, cervical cancer, and prostate cancer [81,84,85,86,87,90,95,97,105].

PDCD10 is also associated with maintaining the stemness of cancer stem cells. By analyzing the expression of miRNAs, Feng et al. showed a downregulation of miR-200c in breast cancer stem cells compared with breast cancer MCF-7 cells [88]. In vitro and in vivo experiments demonstrated that miR-200c can promote the self-renewal and growth of breast cancer stem cells. PDCD10 was identified as the target of miR-200c and overexpression of PDCD10 rescued the oncogenesis inhibited by miR-200c, indicating that PDCD10 exerted a pro-tumorigenic role in breast cancer by maintaining the stemness of tumor stem cells [88].

#### 3.1.2. PDCD10 Promotes Tumor Migration, Invasion, and Metastasis

More than 90% of cancer-related deaths are associated with tumor metastasis [106]. In order to disseminate from the primary site, tumor cells deriving from the epithelium must become migratory and invasive [107]. The PDCD10 expression level was reported to be associated with tumor stage and nodal involvement in various cancer types [85,95,104], suggesting that PDCD10 may promote tumor metastasis. Evidence from in vivo and in vitro experiments supports this point. For example, as the target of miR-103, overexpression of PDCD10 rescued the cellular migration and invasion inhibited by miR-103 in A549 cells (human NSCLC cell line) [85]. Epithelial-mesenchymal transition (EMT) is a key process during tumor invasion and metastasis [108]. Increased PDCD10 has been demonstrated to be able to induce EMT in HCC, breast cancer, prostate cancer, ovarian cancer, and pituitary adenomas [29,77,87,90,96]. The underlying mechanism by which PDCD10 regulates the EMT process in cancers may be mediated by the nuclear translocation of YAP and the downregulation of Rho/ROCK signaling [29,77].

During migration, cells can adopt two different movement patterns: mesenchymal and amoeboid [109]. The GCKIII kinases regulated by PDCD10 have recently been demonstrated to determine the mode of cancer cell migration and metastasis [78]. High expression of PDCD10 in cancer cells promotes the recruitment of STK24&26 to sites of actomyosin, which in turn facilitates the three-dimensional migration of tumor cells through confined environments, via coupling the actomyosin network to the plasma membrane and driving cell contraction, ultimately leading to tumor metastasis [78].

During tumor development and metastasis, glucose metabolism is also essential to fulfill the high energy requirements of tumors. Notably, a recent study reported that PDCD10 can increase the activity and level of LDHA and promote the glycolysis of prostate cancer cells, suggesting its regulatory role in tumor metabolism [87].

### 3.2. PDCD10 Exerts Anti-Tumorigenic Effects

#### 3.2.1. PDCD10 Depletion Is Associated with Meningioma

In 2013, Riant et al. showed for the first time that the PDCD10 mutation was specifically associated with multiple meningiomas in CCM patients [98]. The results of subsequent studies also supported this association [99,100], suggesting that PDCD10 depletion may be responsible for the oncogenesis of meningiomas. However, it is currently not clear whether the PDCD10 mutation is a cause of meningioma or not, but if it is a cause of meningioma, how mutant PDCD10 leads to meningioma formation is worth exploring. Interestingly, this association was not observed in patients with CCM1 or CCM2 mutations [98]. Thus, dysfunctional CSC may not be the primary instigator in meningiomas with mutant PDCD10.

#### 3.2.2. Loss of PDCD10 Promotes Tumor Metastasis by Activating CAFs

CAFs have been demonstrated to play multiple roles in the oncogenesis and progression of tumors by secreting inflammatory ligands, growth factors, and extracellular matrix proteins [110]. PDCD10 in CAFs was reported to regulate YAP/TAZ activation negatively in a non-canonical pathway independently of Hippo signaling, the STRIPAK complex, and CSC [5]. Loss of PDCD10 in CAFs drives the activation of CAFs, promotes the nuclear translocation of YAP, and consequently leads to the metastatic dissemination of tumors through extracellular matrix remodeling in mouse models of breast cancer. Mechanistically, as a binding partner of paxillin, PDCD10 competitively inhibits the interaction between FAK and paxillin, reducing FAK/Src activation, actomyosin coupling, traction forces on the extracellular matrix, and focal adhesion signaling. Loss of PDCD10 in CAFs remodels the extracellular matrix network and eventually causes changes in tumor stiffness and increased metastatic spread, via a positive feedback loop between mechanotransduction and YAP signaling [5].

#### 3.2.3. Loss of PDCD10 Promotes Chemoresistance of Tumors

Chemoresistance in cancer cells is a major cause of treatment failure and tumor recurrence. Zhang et al. reported for the first time that PDCD10 was reduced in chemo-resistant colorectal cancer cells compared with chemo-sensitive cells, while overexpression of PDCD10 restored the sensitivity of colorectal cancer cells to 5-fluorouracil and oxaliplatin [91]. Its effect on colorectal cancer was also further confirmed by another study [92]. Similarly, loss of PDCD10 also enhanced the chemoresistance of tumor cells in breast cancer and glioma [97,101].

#### 3.2.4. Loss of PDCD10 Promotes Angiogenesis of Tumors

Hypoxia is a common feature of various solid cancers due to over-exuberant tumor cell proliferation. In order to improve impaired energy metabolism caused by hypoxia, the tumor usually induces the expression of some hypoxia-related molecules to promote angiogenesis. In retinoblastoma, hypoxia increased miR-181b levels in a HIF-1α-independent manner. As a downstream target of miR-181b, PDCD10 was deemed to be responsible for miR-181b-mediated angiogenesis. Downregulation of PDCD10 in tumor cells promoted the capillary tube formation of human umbilical vein endothelial cells and tumor angiogenesis [89]. Likewise, loss of PDCD10 is also associated with hyper-angiogenesis in glioblastoma (GBM), as discussed below.

### 3.3. The Controversy Regarding the Role of PDCD10 in Gliomas

Glioma is the most common primary central nervous system tumor in adults and accounts for approximately 80% of all malignant brain tumors, among which GBM, the most malignant glioma, contributes approximately 70% of all gliomas [111]. As previously mentioned, the depletion of PDCD10 in endothelial cells contributes to CCM lesions. Another report showed that CCM lesions were also associated with neuroglia (the cell of origin of glioma), in which loss of PDCD10 promoted cell survival, cell proliferation, and vascular lesions [112]. Similar to the function of PDCD10 in CCM, PDCD10 deficiency seems to promote the cell proliferation, migration, and angiogenesis of glioma. Lambertz et al. reported for the first time that PDCD10 expression was downregulated in GBM, which was associated with an increased proliferation of tumor cells [113]. Mechanistically, GBM cells with a PDCD10 deficiency increased the expression of EphB4 and activated the Erk1/2 pathway, which promoted malignant GBM behaviors 102]. Additionally, the knockdown of PDCD10 in tumor cells also facilitated angiogenesis in a mice GBM xenograft model [102,113]. Interestingly, loss of PDCD10 in endothelial cells was also found to promote GBM growth 103], suggesting a bidirectional dependency between tumor cells and endothelial cells. The crosstalk may be mediated by some extracellular proangiogenic factors, such as VEGF [103].

However, a recent study reported that PDCD10 actually exerted a pro-tumorigenic effect in GBM [94]. In addition to tumor cells themselves, tumor growth also depends on the diversity of cell types in tumor tissue. The tumor microenvironment (TME) comprises an extracellular matrix, soluble products, and various non-cancerous cells including immune cells [114]. The crosstalk between immune cells and cancer cells in TME determines tumor growth and metastasis. For example, the M2-phenotype of macrophages usually performs an anti-inflammatory function to induce the immune escape of tumors, which in turn promotes tumor progression [115]. Because the nude mice xenograft model (rather than animal model) with a healthy immune function was utilized, the role of tumor-associated immune cells could not be properly evaluated in the above studies [101,102,103]. Zhang et al. reported that the overexpression of PDCD10 in GBM cells promoted the recruitment and M2 polarization of glioma-associated microglia/macrophages via CXCL2-CXCR2 paracrine signaling, which contributed to tumor growth in vivo [94]. Consistent with the results, data from bioinformatics databases also revealed that PDCD10 was upregulated in patients with GBM and significantly correlated with poor prognosis [94].

Thus, to figure out the precise role of PDCD10 in gliomas, more research in huge clinical cohorts is encouraged in the future.

## 4. Perspective

Due to its functional complexity, PDCD10 exhibits different effects in cancers. As an adaptor protein, the function of PDCD10 mainly depends on its interactors in different cell types. In addition to these interactors in cancers (which were discussed above), others may also be involved in the regulation of tumor development and progression mediated by PDCD10. For example, given the interaction between PIP3 and PDCD10, we speculate that PDCD10 may competitively inhibit the interaction between PIP3, PDK1, and Akt, which in turn suppresses the activation of the Akt pathway. Consistent with this possibility, the expression of PDCD10 in GBM is negatively correlated with the activation of Akt [113]. Moreover, activating the mutation of PIK3CA and AKT1 in animal models and CCM patients resulted in CCM lesions similar to PDCD10 depletion [116,117]. Thus, the PI3K-Akt pathway may also be one of the keys to the negative regulation of tumorigenesis mediated directly by PDCD10 in brain tumors, though further investigation is needed. In endothelial cells, PDCD10, together with CCM1 and CCM2, form the CSC complex to exert function. The dysfunctional CSC complex contributes to CCM lesions. Recently, several studies also investigated the roles of CCM1 and CCM2 in cancers. For example, CCM1 was reported to act as an anti-tumor protein in melanoma [118], but it can also promote the metastasis of prostate cancer by activating YAP/TAZ signaling [119]. In contrast, CCM2 is thought to suppress tumors by mediating TrkA-dependent death in medulloblastomas and neuroblastomas [120]. Notably, a recent study systematically analyzed the expression of CCM1, CCM2, and CCM3 in various types of cancers, indicating that differential expression patterns of the CSC may be associated with certain types and grades of cancers [121]. Due to the effects of CSC on cell survival, migration, and cell junction, the roles of CSC in cancer require intensive investigation in the future.

On the other hand, based on its structure, PDCD10 may link the interactors binding to the FAT-Homology domain (such as paxillin and CCM2) to the interactors binding to the dimerization domain (GCKIII kinases), which suggests that paxillin/CSC can be regulated by GCKIII kinases. Although the interactors binding to the FAT-Homology domain of PDCD10 (STRNs, paxillin, CCM2, etc.) are mutually exclusive, homodimerized PDCD10 may link them to each other, which indicated that paxillin and CSC may be recruited and regulated by the STRIPAK complex. However, the function of the homodimerization of PDCD10 is currently unclear in cancers.

Few studies have focused on the effects of PDCD10 on the immune system. However, Zhang et al. reported that the PDCD10-STK24 complex was involved in the regulation of neutrophil degranulation during acute innate immune responses [50]. In cancers, PDCD10 was also found to promote the recruitment and M2 polarization of glioma-associated micro-glia/macrophages [94]. Additionally, the role of PDCD10 in the modulation of immune cells in cancer is also emerging in other cancer types. In cholangiocarcinoma, the expression of PDCD10 was significantly correlated with the infiltration level of nine immune cell types [104]. Mucosal-associated T cells, NK cells, and monocytes were negatively correlated with PDCD10 expression, while B cells, dendritic cells, CD4+ T cells, iTregs, nTregs, and Tr1s were positively correlated, suggesting that PDCD10 may promote the formation of the suppressive immune microenvironment in cholangiocarcinoma [104]. As a protein ubiquitously expressed in various tissues and cell types, the role of PDCD10 in non-neoplastic cells in TME remains unclear. Therefore, the relationship between PDCD10 and TME also deserves further exploration.

Regardless of the dual role of PDCD10 in oncogenesis, developing drugs that target PDCD10 in specific tumor types is still a promising perspective for cancer therapy. On the other hand, multiple miRNAs have been identified as upstream regulators of PDCD10 expression (Table 1). Targeting these regulators of PDCD10 also seems to be an efficient strategy for the treatment of cancers.

## 5. Conclusions

In this review, we provided an overview of the structure, interactors and regulators of PDCD10, and summarized the molecular functions of PDCD10 in cancers. As an adaptor protein, PDCD10 seems to have a dual role (either pro- or anti-tumor effects) in various cancer types, which may depend on cell/tissue specificity with different cellular interactors. To figure out what exactly determines different functions of PDCD10 in different cancers, more studies are needed in the future. Moreover, designing and developing effective inhibitors of PDCD10 will be a promising treatment strategy for some cancer types. 

## Figures and Tables

**Table 1:** MicroRNAs targeting PDCD10.

MicroRNA	Disease/Cell	Function by Targeting PDCD10	Upstream Regulators	Ref
miR-1-3p	HEK293 cell	associated with cell death induced by ochratoxin A	/	[40]
miR-107	Mesenchymal stem cell	promotes cell survival during ischemic preconditioning	HIF-1α	[41]
miR-107	Alzheimer’s disease	attenuates neurotoxicity	/	[15]
miR-495	Ankylosing spondylitis	involved in pathogenesis of ankylosing spondylitis	/	[16]
miR-613	Myocardial ischemia/reperfusion injury	suppresses ischemia-reperfusion-induced cardiomyocyte apoptosis	/	[14]
miR-26-5p	Bladder cancer	inhibits the tumor	/	[84]
miR-103	Non-small cell lung cancer	inhibits cell proliferation, migration, and invasion	/	[85]
miR-103	Prostate cancer	inhibits cell proliferation and invasion	/	[86]
miR-432	Prostate cancer	inhibits proliferation, metastasis, and glycolysis	CircSMARCA5	[87]
miR-200c	Breast cancer stem cell	inhibits self-renewal of stem cell	/	[88]
miR-181b	Retinoblastoma	promotes angiogenesis induced by hypoxia	/	[89]
miR-222-3p	Ovarian cancer	inhibits EMT	SNAI2	[90]
miR-425-5p	Colorectal cancer	enhances chemoresistance	/	[91]
miR-46146	Colorectal cancer	enhances chemoresistance	/	[92]

**Table 2:** The dual role (pro- or anti-tumor effects) of PDCD10 in cancers.

**Pro-Tumorigenic Effects of PDCD10**			
**Cancer/Cell Types**	**Interactions/Pathways**	**Effects**	**Ref**
Lymphomas	-	promoted proliferation and decreased apoptosis	[93]
Glioma	CXCL2-CXCR2	promoted recruitment and M2 polarization of glioma-associated microglia/macrophages	[94]
Non-small cell lung cancer	-	promoted cell proliferation and migration	[85]
Breast cancer	TRIM59; STK24&26; Rho/ROCK signaling	promoted survival, stemness, EMT, and metastasis of tumor cells	[29,78,88]
Liver cancer	PP2A-YAP signaling	promoted cell proliferation, migration, invasion/metastasis, and EMT	[77]
Ovarian cancer	ERK signaling; RhoA signaling; β-catenin signaling	promoted cell proliferation, migration, and EMT	[90,95]
Prostate cancer	-	Promoted cell proliferation, migration, EMT, and glycolysis	[86,87]
Bladder cancer	-	promoted cell proliferation	[84]
Pituitary adenomas	CXCR2-Akt/Erk signaling	promoted cell proliferation, migration, and invasion	[96]
Cervical cancer	-	promoted chemoresistance of tumor cells	[97]
**Anti-Tumorigenic Effects of PDCD10**			
**Cancer/Cell Types**	**Interactions/Pathways**	**Effects**	**Ref**
Meningioma	-	PDCD10 deletion was specifically associated with multiple meningiomas	[98,99,100]
Glioma	Soluble factors; EphB4- Erk1/2	Knockdown of PDCD10 facilitated tumor cell proliferation, migration, invasion, and stimulated angiogenesis.	[101,102]
Retinoblastoma	-	Downregulation of PDCD10 in retinoblastoma cells promoted angiogenesis	[89]
Colorectal cancer	-	Loss of PDCD10 enhanced chemoresistance of tumor cells	[91,92]
Breast cancer	-	Loss of PDCD10 enhanced chemoresistance of tumor cells	[97]
Endothelial cells	Growth factors signaling	Knockdown of endothelial PDCD10 stimulated angiogenesis and tumor growth of glioma	[103]
Cancer-associated fibroblasts	Paxillin	Loss of PDCD10 in CAFs drove remodeling of extracellular matrix network and increased metastatic spread of tumor	[5]

## Abbreviations

PDCD10: programmed cell death 10; TFAR-15: TF-1 cell apoptosis related gene-15; FAT: focal adhesion targeting; CCM3: Cerebral cavernous malformation 3; CCM: cerebral cavernous malformation; CSC: CCM signaling complex; GCKIII: germinal center kinase III; STRNs: striatins; PIP3: phosphatidylinositol-3,4,5-trisphosphate; STRIPAK: striatin-interacting phosphatase and kinase; PP2A: protein phosphatase 2A; ERM: ezrin/radixin/moesin; CAFs: cancer-associated fibroblasts; HCC: hepatocellular carcinoma; TRIM: tripartite motif; miRNAs: microRNAs; NSCLC: non-small cell lung cancer; EMT: Epithelial-mesenchymal transition; GBM: glioblastoma; TME: tumor microenvironment.
